# Micellization Behaviour of Linear and Nonlinear Block Copolymers Based on Poly(n-hexyl isocyanate) in Selective Solvents

**DOI:** 10.3390/polym12081678

**Published:** 2020-07-28

**Authors:** Aggelos Vazaios, Athanasios Touris, Mikel Echeverria, Georgia Zorba, Marinos Pitsikalis

**Affiliations:** Industrial Chemistry Laboratory, Department of Chemistry, National and Kapodistrian University of Athens, Panepistimiopolis Zografou, 15771 Athens, Greece; aggelosvazaios@gmail.com (A.V.); atouris31@gmail.com (A.T.); mechmug@gmail.com (M.E.); gzormpa@hotmail.com (G.Z.)

**Keywords:** micelles, self-assembly, solution properties, polyisocyanates, rod-coil copolymers, light scattering, atomic force microscopy

## Abstract

Block copolymers have attracted significant scientific and economic interest over the last decades due to their ability to self-assemble into ordered structures both in bulk and in selective solvents. In this work, the self-assembly behaviour of both linear (diblocks, triblocks and pentablocks) and nonlinear (miktoarm stars and a block-graft) copolymers based on poly(n-hexyl isocyanate), PHIC, were studied in selective solvents such as n-heptane and n-dodecane. A variety of experimental techniques, namely static and dynamic light scattering, dilute solution viscometry and atomic force microscopy, were employed to study the micellar structural parameters (e.g., aggregation number, overall micellar size and shape, and core and shell dimensions). The effect of the macromolecular architecture, the molecular weight and the copolymer composition on the self-assembly behaviour was studied. Spherical micelles in equilibrium with clusters were obtained from the block copolymers. Thermally stable, uniform and spherical aggregates were found from the triblock copolymers. The poly(n-hexyl isocyanate)-b-polyisoprene-b-poly(n-hexyl isocyanate),-HIH copolymers tend to adopt closed loop conformation, leading to more elongated cylindrical-type structures upon increasing the concentration. Clustering effects were also reported in the case of the pentablock terpolymers. The topology of the blocks plays an important role, since the poly(n-hexyl isocyanate)-b-polystyrene-b-polyisoprene-b-polystyrene-b-poly(n-hexyl isocyanate), HSISH terpolymer shows intermicellar fusion of spherical micelles, leading to the formation of extended networks. The formation of spherical micelles in equilibrium with clusters was obvious in the case of the miktoarm stars, whereas the block-graft copolymer shows the existence of mainly unimolecular micelles.

## 1. Introduction

Block copolymers are without any doubt the most important class of polymeric materials [1,2]. This is due (a) to the numerous methodologies and techniques that are available for the synthesis of well-defined products with predetermined molecular characteristics and low molecular and chemical heterogeneity. A huge variety of structures with all kinds of combinations of polymeric chains, molecular weights and compositions has been synthesized over the years [3,4], (b) to their self-assembly behaviour as a result of the immiscibility of the constituent blocks. In bulk, this immiscibility results in microphase separation and the formation of long-range ordered nanostructures, such as spheres, cylinders, lamellae, bicontinuous structures, etc. [5,6]. In a selective solvent, which is a thermodynamically good solvent for the one block and non-solvent for the other block, the copolymers self-assemble, leading to the formation of supramolecular structures, called micelles [7,8,9], and (c) to their numerous and diverse applications, such as colloidal stabilization [10,11], latex technology [12], compatibilization in polymer blends [13], controlled drug delivery [14,15,16,17], water purification [18,19], viscosity and surface modification [20,21,22,23], catalytic supports [24], lithographic templates [25], nanoreactors [26,27], etc.

More recent advances in polymer chemistry have allowed the synthesis of nonlinear copolymer structures in a very controlled way [28,29,30,31,32]. Thus, star-block, miktoarm star, graft, and block-graft copolymers along with more complex macromolecular structures have been synthesized. From these studies, it became evident that the polymer properties and especially their self-assembly behaviour both in bulk and in selective solvents is significantly influenced by the macromolecular architecture [33,34,35,36,37,38].

In the past, a great effort was made by our group towards the synthesis of well-defined products based on poly(hexyl isocyanate), PHIC. PHIC chains are very stiff adopting dynamic helical structures both in bulk and in a wide range of solvents [39,40,41,42]. The reason for this behaviour is the resonance delocalization of the nitrogen and carbonyl electrons and the steric hindrance induced by the monomer’s bulky side group. Thus, polyisocyanates possess an extended 8/3 helical conformation instead of a restricted coplanar conformation [43]. Block copolymers with polystyrene (PS) and polyisoprene (PI) [44,45,46], triblock copolymers, pentablock terpolymers [47], graft copolymers, star polymers, star-block copolymers, miktoarm stars and block-graft copolymers [48,49,50] have been synthesized based either on anionic [51] or coordination polymerization of n-hexyl isocyanate, HIC employing half-Titanocene complexes [52,53,54,55,56,57]. Polyisocyanates have found tremendous applications in nanoscience and materials science as degradable and chiral recognition materials, optical switches, liquid crystals, etc. [58,59,60,61,62,63].

**Scheme 1:**
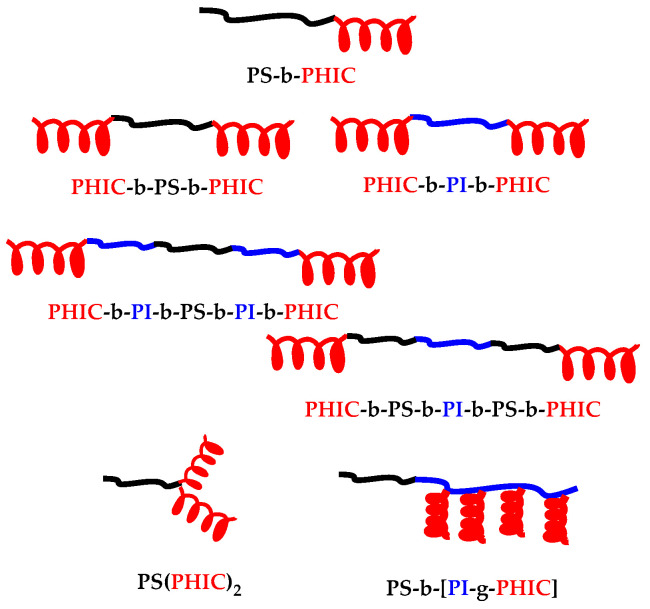
Linear and nonlinear poly(n-hexyl isocyanate) (PHIC)-based block copolymers.

In this work, the micellization behaviour of certain PHIC-based polymers will be examined by a variety of experimental techniques, such as low-angle laser light scattering (LALLS), dynamic light scattering (DLS), dilute solution viscometry, along with atomic force microscopy (AFM). In particular, these structures involve PS-b-PHIC diblock copolymers, PHIC-b-PS-b-PHIC and PHIC-b-PI-b-PHIC triblock copolymers, PHIC-b-PI-b-PS-b-PI-b-PHIC and PHIC-b-PS-b-PI-b-PS-b-PHIC pentablock terpolymers, PS(PHIC)_2_ miktoarm stars and a PS-b-(PI-g-PHIC)block-graft terpolymer (Scheme 1). Previous studies have focused mainly on diblock and triblock copolymers containing PHIC chains, showing unique characteristics on the self-assembly properties and a tremendous influence of the rod-like nature of polyisocyanates [39,64,65,66,67]. Therefore, cylindrical micelles or vesicular structures were obtained reflecting the liquid crystalline properties of the PHIC chains. The micellization behaviour of rod-coil copolymers was not studied in depth as in the case of coil-coil copolymers. The parameter of macromolecular architecture is a new feature, which dramatically affects the self-assembly behaviour of rod-coil copolymers in selective solvents.

## 2. Materials and Methods

### 2.1. Materials

n-Heptane and n-dodecane used for the characterization were refluxed from calcium hydride and distilled before use. Tetrahydrofuran (THF) was refluxed over metallic sodium and distilled before use.

### 2.2. Polymer Synthesis

The polymer synthesis was accomplished through anionic and coordination polymerization techniques as previously described in the literature [44,45,46,47,48,49,50].

### 2.3. Characterization Techniques

Size exclusion chromatography (SEC) experiments were conducted at 40 °C using a modular instrument consisting of a Waters Model 510 pump (Waters, Milford, MA, USA), a Waters Model U6K sample injector (Waters, Milford, MA, USA), a Waters Model 401 differential refractometer Waters, Milford, MA, USA), a Waters Model 486 UV spectrophotometer (Waters, Milford, MA, USA) and a set of 4 μ-Styragel columns with a continuous porosity range from 500 A to 10^5^ A. The columns were housed in an oven at 25 °C. THF was the carrier solvent at a flow rate of 1 mL/min.

Low-angle laser light scattering measurements were performed with a Chromatix KMX-6 photometer (Milton Roy, LDC Division, Riviera Beach, FL, USA) at 25 °C equipped with a 2 mW He-Ne laser operating at λ = 633 nm. Equation (1) describes the concentration dependence of the reduced intensity:

(1) $$\frac{Kc}{\Delta R_{\theta }}=\frac{1}{\bar{M_{w}}}+2A_{2}c+\ldots$$

where *K* is a combination of optical and physical constants, including the refractive index increment, *dn/dc*, and the excess Rayleigh ratio of the solution over that of the solvent, Δ*R_θ_*. Stock solutions were prepared, followed by dilution with the solvent to obtain appropriate concentrations. All solutions and solvents were optically clarified by filtering through 0.22-μm pore size nylon filters directly into the scattering cell.

Refractive index increments, *dn/dc*, at 25 °C were measured with a Chromatix KMX-16 (Milton Roy, LDC Division, Riviera Beach, FL, USA) refractometer operating at 633 nm and calibrated with aqueous NaCl solutions.

Dynamic light scattering measurements were conducted with a Series 4700 Malvern system (Grovewood Road. Malvern, UK) composed of a PCS5101 goniometer with a PCS stepper motor controller, a Cyonics variable power Ar^+^ laser operating at 488 nm, a PCS8 temperature control unit, a RR98 pump/filtering unit and a 192 channels correlator for the accumulation of the data. The correlation functions were analysed by the cumulant method and the CONTIN software [68]. Measurements were carried out at 45°, 90° and 135°. The angular dependence of the ratio Γ/q^2^, where Γ is the decay rate of the correlation function and q is the scattering vector, was not very important for most of the micellar solutions. In these cases, apparent translational diffusion coefficients at zero concentration, $D_{0,\;app}$, were measured using Equation (2):

(2) $$D_{app}=D_{0,\;app}\left(1+k_{D}c\right)$$

where *k_D_* is the coefficient of the concentration dependence of the diffusion coefficient. Apparent hydrodynamic radii at infinite dilutions, *R_h_*, were calculated by aid of the Stokes–Einstein Equation (3):

(3) $$R_{h}=kT/6\pi \eta_{s}D_{0,\;app}$$

where *k* is the Boltzmann’s constant, *T* is the absolute temperature and *η_s_* is the viscosity of the solvent.

Viscometric data were analysed using the Huggins Equation (4):

(4) $$\frac{\eta_{sp}}{c}=\left[\eta \right]+K_{H}{\left[\eta \right]}^{2}c+\ldots$$

and the Kraemer Equation (5):

(5) $$\frac{\ln\eta_{r}}{c}=\left[\eta \right]+K_{K}{\left[\eta \right]}^{2}c+\ldots$$

where *η_r_*, *η_sp_* and [*η*] are the relative, specific and intrinsic viscosities respectively, and *K_H_* and *K_K_* are the Huggins and Kraemer constants, respectively. All measurements were carried out at 25 °C. Cannon–Ubbelohde dilution viscometers equipped with a Schott-Geräte AVS 410 automatic flow timer were used. Viscometric radii, *R_v_*, were calculated from Equation (6):

(6) $$R_{v}={\left(\frac{3}{10\pi N_{A}}\right)}^{1/3}{\left(\left[\eta \right]M_{w,\;app}\right)}^{1/3}$$

where $M_{w,\;app}$ is the weight average molecular weight determined by light scattering measurements.

Table 1 H-NMR spectra were recorded in d-chloroform at 305.0 K with a Bruker Avance III 600 NMR spectrometer (Bruker, BioSpin, Rheinstetten, Germany).

Atomic force microscopy (AFM) was performed with a Nanoscope IIIa Microscope with a multimode controller (Veeco Instruments, Plainview, NY, USA) at ambient temperature. Drop cast films were prepared on a freshly prepared mica surface from a sample concentration of 1 mg mL^−1^ in suitable solvent. The tapping mode was employed with an antimony-doped Si tip (radius < 10 nm) at a line scanning frequency of 1 Hz.

## 3. Results

### 3.1. Micellization Behavior of PS-b-PHIC, SH Block Copolymers in n-Heptane

The diblock copolymers having polystyrene (PS) and PHIC blocks were synthesized either by anionic (SH121/90/10 and SH187/92/8) or by coordination polymerization techniques (SH14/3/97, SH7.5/9/91, SH19.5/11/89 and SH14/18/82) [44,45,46,47,48,49,50]. The samples are symbolized as SH followed by numbers denoting the sample’s molecular weight (in KDa), the % wt content is PS and the % wt content in PHIC. The molecular characteristics are given in Table 1. The micellar solutions were obtained in n-heptane, which is a selective solvent for the PHIC block. In other words, the micelles consist of a core from the amorphous and glassy PS chains and a soluble corona from the rod-like PHIC chains. The samples with the rather small amount of PS were directly dissolved in the selective solvent. The dissolution was facilitated by heating the n-heptane solution at 60 °C under argon atmosphere overnight. Taking into account the low molecular weight of the PS blocks and their subsequently lower Tg values, this thermal treatment is able to provide enough mobility to the PS blocks in order to achieve the formation of equilibrium micellar structures. The last two samples with the highest PS content were not directly soluble in the selective solvent. In this case, the micellization was promoted by dissolving the block copolymers in a mixture of the selective solvent along with the minimum amount of the common good solvent THF. In this mixture, the block copolymers are readily dissolved and gradual evaporation of the volatile THF leaves stable micellar solutions with the characteristic blue tint, which ensures the formation of large supramolecular structures. All micellar solutions were stable over a period of several weeks.

**Figure 1:**
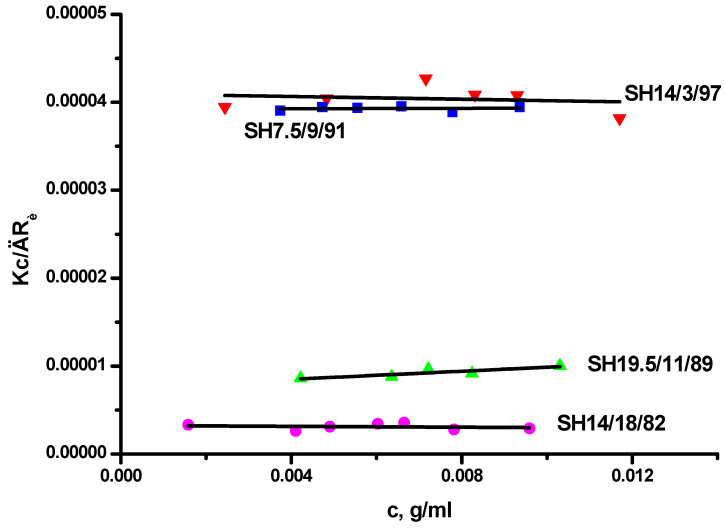
LALLS plots of SH block copolymers in n-heptane.

The LALLS data of the linear SH block copolymers in n-heptane are given in Table 1, whereas characteristic plots are provided in Figure 1. The weight average degree of association, N_w_, defined as the ratio of M_w_ measured in the selective solvent over M_w_ of the diblock in the common good solvent THF, increases upon increasing the PS content. Sample SH14/3/97 with only 3% wt PS leads to almost unimolecular micelles with an average degree of association, N_w_, equal to 1.4. Going to the extreme case, where PS dominates within the copolymer structure (samples SH121/90/10 and SH187/92/8 containing more than 90% PS), the N_w_ values increase dramatically to more than 1300. The LALLS plots for samples SH14/3/97, SH7.5/9/81, SH19.5/11/89 and SH14/18/82 are linear over the concentration range examined, i.e., the critical micelle concentration (CMC) is much lower than the lowest experimentally accessible concentration (approximately 2 × 10^−3^ g/mL), and stable micellar structures exist in that concentration regime. Samples SH121/90/10 and SH187/92/8 show stable micellar structures at concentrations higher than 1.5 × 10^−5^ g/mL. At lower concentrations, much lower apparent molecular weights were obtained, indicating the ongoing association process. However, even for these two samples, it was not possible to identify the CMC values by LALLS measurements. The LALLS plots for these samples are incorporated in the Supplementary Materials Section, SMS. The second virial coefficients are very low, even in the cases of micelles with low N_w_ values, indicating weak interactions of the supramolecular species and the selective solvent.

The DLS data of block copolymers in n-heptane are given in Table 1. The plots are linear (characteristic examples are given in the SMS) but with very low k_D_ values. This result is in agreement with the low A_2_ values obtained in the selective solvent, since k_D_ and A_2_ are related through the following equation:

k_D_ = 2A_2_M + k_f_ − u (7)

where M is the molecular weight, k_f_ is the coefficient of the concentration dependence of the friction coefficient and u is the partial specific volume of the polymer. Due to the similar molecular weights in both solvents, THF and n-heptane, a decrease in A_2_ is accompanied by a decrease in k_D_ as well. CONTIN analysis provided suitable insight into the self-assembly process and the formation of the supramolecular structures.

Sample SH14/3/97, with the lowest PS content, forms very small spherical structures, as revealed by their low size along with the absence of any angular dependence in DLS measurements. The polydispersity factor μ_2_/Γ^2^, where Γ is the decay rate of the correlation function and μ_2_ is the second moment of the cumulant analysis, is always lower than 0.1, indicating the presence of monodisperse small micellar structures.

CONTIN analysis for samples SH7.5/9/81, SH19.5/11/89 and SH14/18/82 revealed the presence of bimodal distributions, indicating the presence of higher-order equilibrium in solution. Judging from the relative R_h_ values of the different populations, it is obvious that equilibrium between micelles and clusters exist. For samples SH19.5/11/89 and SH7.5/9/91, the clusters are the minority component (less than 10% by intensity), but for sample SH14/18/82, the contribution of the clusters is much more pronounced (higher than 30% by intensity). In all cases, however, the micelles are spherical (there is no angular dependence) and thermally stable up to 55 °C. In addition, the micelles are almost monodispersed, taking into account the lower than 0.1 values of the polydispersity factor μ_2_/Γ^2^.

Samples SH121/90/10 and SH187/92/8 with the highest N_w_ values show unimodal distributions in CONTIN analysis. As revealed in the other cases, the micelles are spherical and monodispersed with polydispersity factor values μ_2_/Γ^2^ of 0.05. They are also thermally stable up to 55 °C. Despite the very high N_w_ values of samples SH121/90/10 and SH187/92/8, their R_h_ values are not so high, indicating that the micelles are very compact and behave more or less as hard spheres.

The thermal stability of all micelles can be explained considering that the core material is the amorphous and glassy PS with high Tg value and, more importantly, that the corona material is the stiff and helical PHIC, which does not appreciably change conformation at the temperature range examined in this work.

Several theoretical models have been proposed to describe the micelle structure. The case of micelles consisting of small cores and large coronas can be well described by the star model. If N_A_ and N_B_ are the lengths of the corona and core-forming blocks A and B, respectively, the star model will be valuable when N_A_ >> N_B_. According to the Halperin’s star model [69], the following scaling relationships between the parameters of interest for block copolymers can be obtained:

*Aggregation number N~N_B_^4/5^*

*Core radius R_c_~N_B_^3/5^*

*Micelle radius R~N_B_^4/25^ N_A_^3/5^*

The other limiting case is the situation of micelles consisting of a large core and a relatively thin corona, i.e., when N_B_ >> N_A_ can be described by mean density models [70,71,72]. According to these models, the volume fraction of the A segments in the corona is assumed to be independent of the distance from the core. The following scaling relationships are obtained:

*Aggregation number N~N_B_*

*Core radius R_c_~N_B_^2/3^*

*Micelle radius R~N_B_^2/3^*

To further test the validity of these models, the radius of the micellar core was calculated using the following equation:

R_c_ = (3M_w,mic_ wt_PS_/4πN_A_ d_PS_ φ_PS_)^1/3^ (8)

where M_w,mic_ is the micellar molecular weight, wt_PS_ is the weight fraction of the core-forming block, d_PS_ is the density of PS and φ_PS_ is the volume fraction of PS in the core. The density of PS is equal to 1.05 (d_PS_ = 1.05). Two limiting cases were considered: the dry core (φ_PS_ = 1) and the swollen core (φ_PS_ = 0.5). The results for both cases are given in Table 2. In addition, the area of the core-corona interface, A_c_, occupied per copolymer chain is given as follows:

A_c_ = 4πR_c_^2^/N_w_ (9)

The A_c_ values for the dry and swollen core cases are also reported in Table 2.

Judging from the high PS content of samples SH121/90/10 and SH187/92/8, it is obvious that the micellar core should be extended and that the supramolecular structures should behave as crew-cut micelles. This assumption was verified by the almost stable values of the ratio R_h_/N_B_^2/3^, the high values of R_c_ compared to the corresponding R_h_ of the micelles and the low A_c_ values considering both the dry and the swollen core cases.

On the other hand, the low PS contents of samples SH14/3/97, SH7.5/9/81, SH19.5/11/89 and SH14/18/82 support the application of the star model for their micellar structures. The R_h_/(N_B_^4/25^ N_A_^3/5^) ratios were, nevertheless, not very stable as would be expected. The reason for this is the fact that there is a small but recorded tendency for the formation of clusters through intermicellar association, which causes deviations from the pure star model, and most importantly that the PHIC corona material is actually very stiff and does not change easily conformations. The star model is more adequate for flexible polymeric chains. However, the relatively low R_c_ and high A_c_ values are more consistent with a star-like structure. These conclusions will be further confirmed by AFM measurements.

### 3.2. Micellization Behavior of PHIC-b-PS-b-PHIC, HSH, Triblock Copolymers in n-Heptane and PHIC-b-PI-b-PHIC, HIH, Triblock Copolymer in n-Dodecane

**Figure 2:**
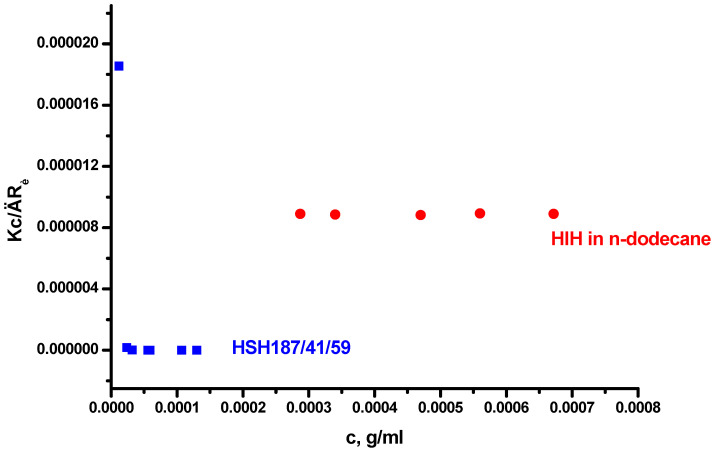
LALLS plots of triblock copolymers HSH#2 in n-heptane and HIH in n-dodecane.

The symmetric triblock copolymers PHIC-b-PS-b-PHIC and PHIC-b-PI-b-PHIC containing PS or polyisoprene (PI) as the middle block were synthesized by anionic polymerization high vacuum techniques [47]. The samples are symbolized as HSH and HIH, respectively. The HSH samples are differentiated by the numbers indicating the sample’s molecular weight (in KDa), the % wt content in PS and the % wt content in PHIC. The molecular characteristics of the samples are given in Table 3. The HSH samples were dissolved in n-heptane, which is selective for the PHIC chains. In order to facilitate the dissolution and achieve equilibrium structures, the solutions were heated at 60 °C overnight in argon atmosphere. PHIC and PI are soluble in n-heptane, and therefore, the HIH copolymer was dissolved in n-dodecane, which is selective for the PI chains. The static and dynamic light scattering data are given in Table 3, whereas characteristic LALLS plots are provided in Figure 2. DLS plots are also given in the SMS. The LALLS plot is linear for HIH-forming structures with a very low N_w_ value. Therefore, almost unimolecular micelles are promoted in n-dodecane. In low concentrations, the formation of unimolecular micelles is favoured due to the rather low PHIC content in the copolymer (16% wt). The linear chains form loops in order to accommodate together the insoluble PHIC chains. This is facilitated by the presence of the flexible and soluble PI block, which is placed at the middle of the structure. These intramolecular associates through loop formation are in equilibrium with intermolecular associates or clusters, leading to higher degrees of aggregation. This equilibrium is shifted towards the clusters upon increasing the concentration. This behaviour has been observed in the case of micellar formation from triblock copolymers in solvents selective for the middle block [73,74,75,76,77].

The HSH samples form multimolecular micelles with N_w_ values equal to 43.5 and 102. The sample with the higher PS content has the higher degree of association, as expected. However, these values are lower than those expected, taking into account the corresponding diblock copolymers, indicating that the splitting of the PHIC chain to two parts surrounding the insoluble corona leads to more soluble supramolecular structures and, therefore, to lower N_w_ values. The LALLS plots are linear for concentrations higher than 10^−5^ g/mL. At lower concentrations, lower apparent molecular weights are observed, verifying that, at this concentration regime, micellar organization takes place. However, the experimental data are not sufficient to provide the CMC value for these triblock copolymers. Finally, the A_2_ values are slightly negative, as a result of the reduced interactions between the triblock copolymer and the selective solvent. The macromolecular architecture definitely plays an important role influencing the micellization behaviour.

The DLS data are displayed in Table 3. Results in the selective solvent and in the common good solvent THF are also provided. The sample HIH revealed bimodal distribution in CONTIN analysis. The major component (approximately 90% by intensity) has slightly lower R_h_ values compared to that measured in THF, confirming the formation of mainly unimolecular micelles in n-dodecane. The second minor population revealed a much higher R_h_ value, which can be attributed to a small amount of multimolecular micelles. These results confirm the previous discussion referring to the presence of equilibrium between intramolecular (loops formation) and intermolecular aggregates (clusters). This conclusion will be further supported by AFM images. The triblock HSH on the other hand showed unimolecular peaks in the CONTIN analysis. The absence of angular dependence and the low polydispersity factor confirms the presence of spherical and monodisperse micellar structures. In addition, these supramolecular structures are thermally stable up to 55 °C. For the HSH triblocks viscometry measurements in dilute solution were conducted as well. The results are given in Table 4, and the viscometry plots are included in the SMS. Very high k_H_ values were obtained as a result of the strong hydrodynamic interactions in solution, as expected in highly associated systems. The viscometric radii, Rv, were in very close agreement with the corresponding R_h_ values, confirming the formation of stable and spherical micelles in n-heptane. The structural characteristics of the HSH micelles are shown in Table 5. Comparing the R_c_ with the corresponding R_h_ values, it is obvious that the cores are not very extended and are relatively compact. The area of the core-corona interface, A_c_, occupied per copolymer chain is much higher in the case of the triblock copolymer micelles than that of the diblock copolymer micelles. This result clearly indicates the effect of the macromolecular architecture on the micellization process.

### 3.3. Micellization Behavior of PHIC-b-PS-b-PI-b-PS-b-PHIC, HSISH, and PHIC-b-PI-b-PS-b-PI-b-PHIC, HISIH, Pentablock Terpolymers in n-Heptane and in n-Dodecane

**Figure 3:**
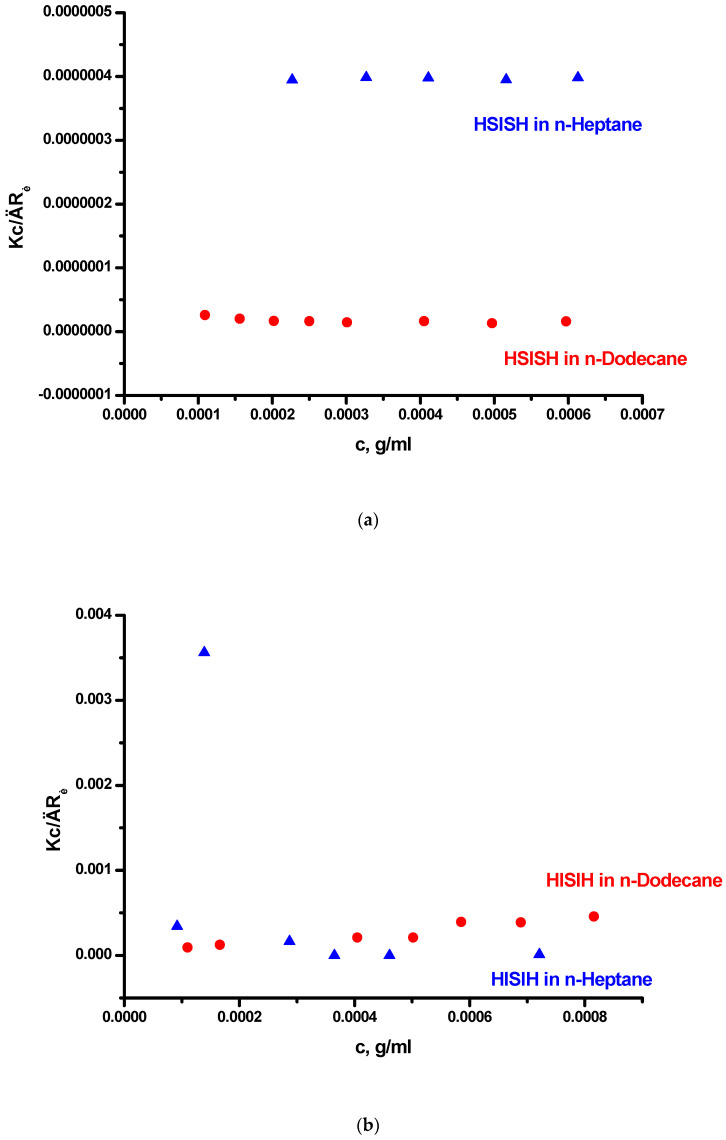
(**a**) LALLS plots of the HSISH pentablock terpolymer in n-heptane and n-dodecane and (**b**) LALLS plots of the HISIH pentablock terpolymer in n-heptane and n-dodecane.

The pentablock terpolymers were synthesized by anionic polymerization and high vacuum techniques [47] and are denoted as HSISH and HISIH. The molecular characteristics of the samples along with their LALLS and DLS data in the common good solvent, THF, and in the selective solvents are given in Table 6. Both solvents are precipitants for the PS chains. On the other hand, n-heptane is a good solvent for PI and PHIC but n-dodecane is a non-solvent for PHIC and PS blocks. The LALLS data for the HSISH in either selective solvent revealed high N_w_ values. The LALLS plots are given in Figure 3a,b. The N_w_ value was considerably higher in n-dodecane than in n-heptane. This difference is reasonable since n-heptane is a good solvent for both PHIC and PI chains, whereas n-dodecane is a good solvent only for the PI chains. The micelles of the pentablocks in n-dodecane should have a similar behaviour with the corresponding HSH triblock copolymers in n-heptane, since in both cases the middle block is insoluble. A peculiar behaviour should be expected in n-heptane, since the insoluble PS blocks are the intermediate blocks in the structure. This may give rise to intermicellar interactions and thus to the formation of more complex supramolecular structures. This will be further confirmed by AFM images.

The pentablock terpolymer HISIH forms aggregates in both solvents. As in the case of the previous terpolymer, the degree of association is higher in n-dodecane than in n-heptane (Figure 3b). However, in both solvents, the N_w_ values are much lower compared to the HISIH terpolymer. This is the result of the different macromolecular architecture. The rearrangement of the blocks within the molecular structure has a tremendous effect on the aggregation behaviour. The insoluble PS block is now located at the middle of the structure and is surrounded by four soluble blocks in n-heptane. The soluble intermediate PI blocks are the minority components interfering the insoluble PS and PHIC blocks, thus restricting the association process and leading to a relatively small degree of association in n-dodecane as well.

**Figure 4:**
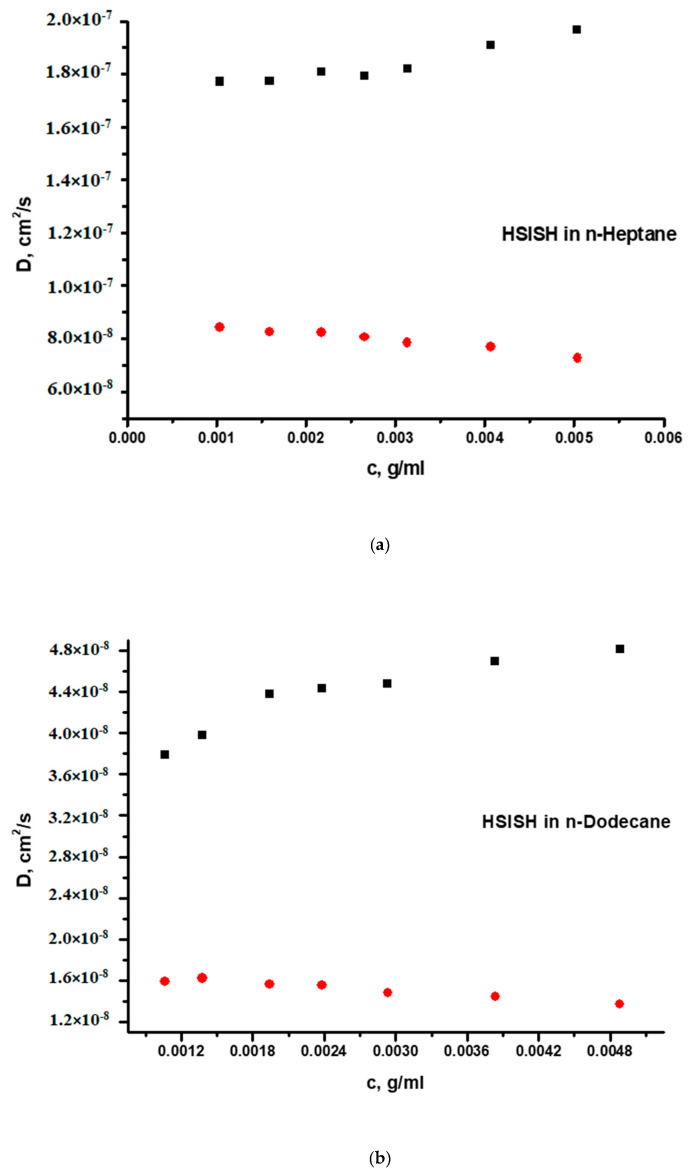
(**a**) DLS plots of the two populations of the HSISH pentablock terpolymer in n-heptane and (**b**) DLS plots of the two populations of the HSISH pentablock terpolymer in n-dodecane.

**Figure 5:**
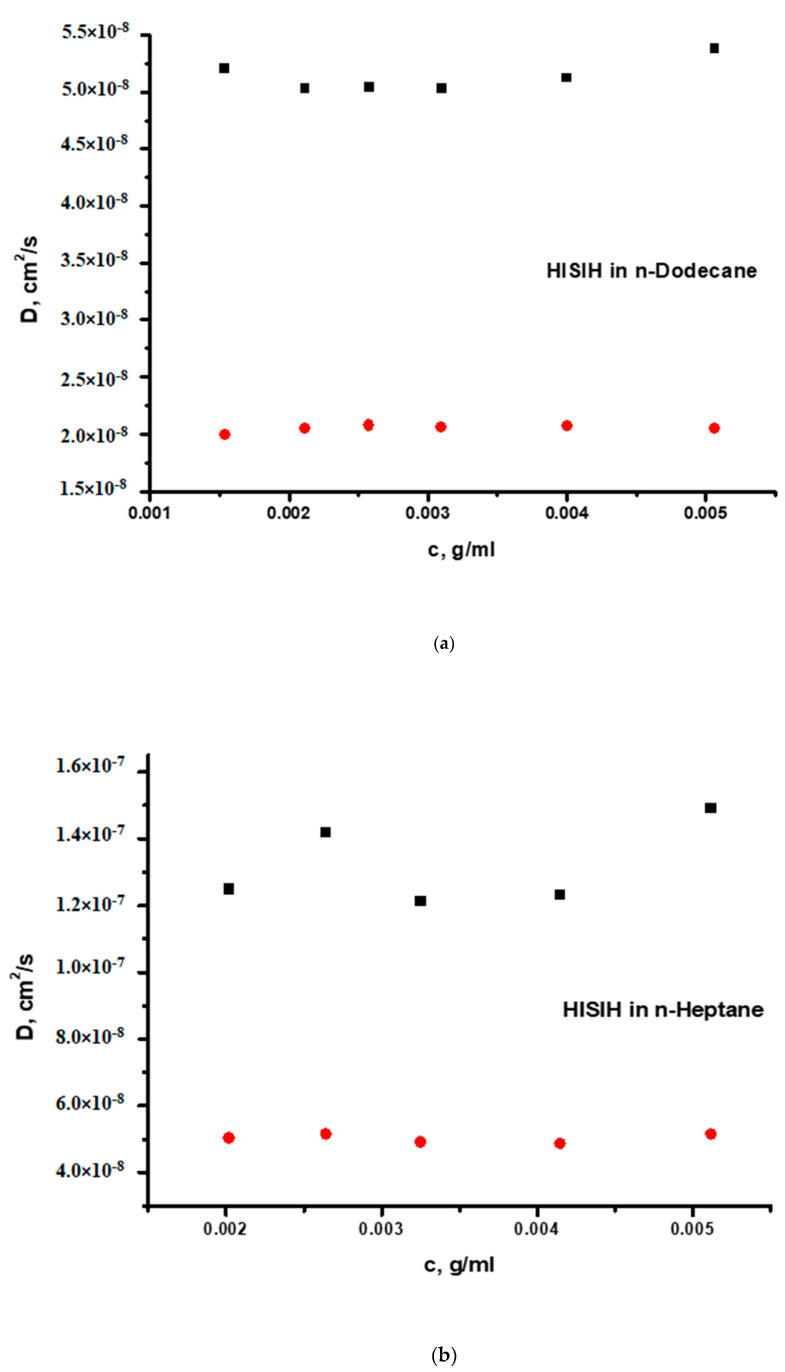
(**a**) DLS plots of the two populations of the HISIH pentablock terpolymer in n-dodecane and (**b**) DLS plots of the two populations of the HISIH pentablock terpolymer in n-heptane.

The DLS measurements reveal a more complex association behaviour in both selective solvents. The DLS plots are provided in Figure 4a,b or Figure 5a,b. It is obvious that, in all cases, there is equilibrium between micellar structures and clusters. This is confirmed by CONTIN analysis showing two respective populations. The first population is angular independent, and the particles have low dispersity (polydispersity factor μ_2_/Γ^2^ < 0.2). On the contrary, the second population is attributed to non-spherical (there is angular dependence) and rather polydisperse particles (μ_2_/Γ^2^ > 0.2) and has a much higher R_h_ values compared to the first population. The k_D_ values were reasonably lower than those measured in the common good solvent THF, and even lower values were observed for the clusters’ populations. Although the HSISH terpolymer has much higher degrees of association, the structures have lower R_h_ values than that of the pentablock HISIH. These results indicate that more compact supramolecular structures are produced from the HSISH terpolymer.

### 3.4. Micellization Behavior of PS(PHIC)_2_ Miktoarm Star Copolymers and PS-b-(PI-g-PHIC) Block Graft Terpolymer in n-Heptane

**Figure 6:**
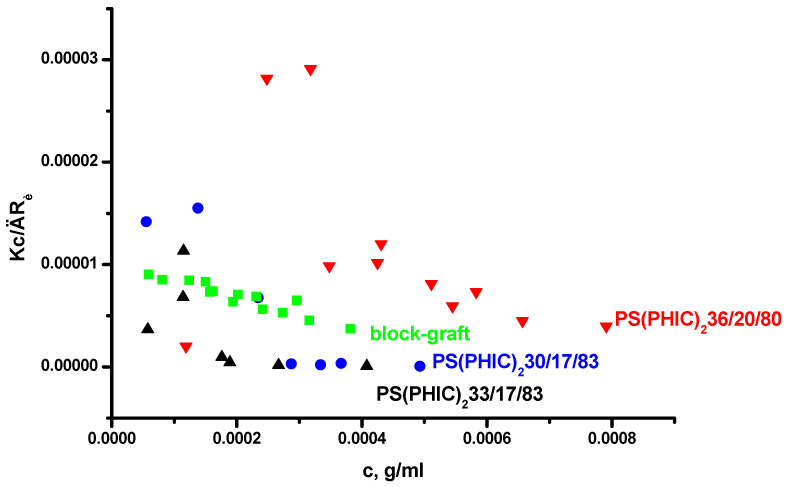
LALLS plots of the miktoarm star and the block-graft copolymers in n-heptane.

**Figure 7:**
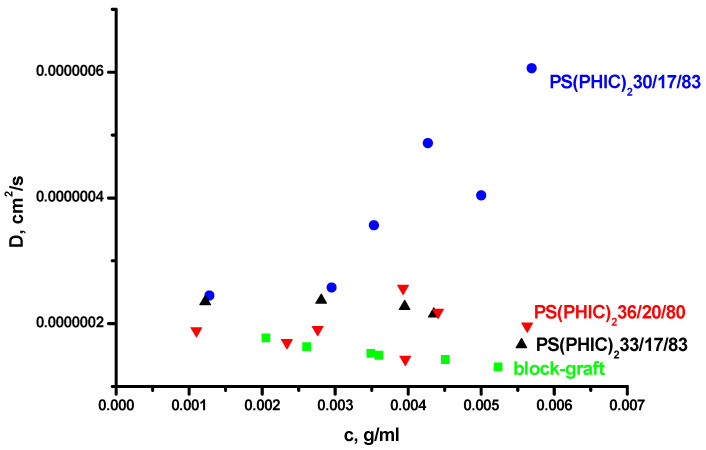
DLS plots of the miktoarm star and the block-graft copolymers in n-heptane.

The PS(PHIC)_2_ miktoarm star copolymers having one PS and two PHIC arms and the PS-b-(PI-g-PHIC) block graft terpolymer were prepared by a combination of coordination and anionic polymerization techniques [49]. The PS arms of the stars and the PS-b-PI backbone of the block-graft terpolymer were synthesized by anionic polymerization, whereas the PHIC chains were synthesized by coordination polymerization. The stars are differentiated by the numbers indicating the sample’s molecular weight (in KDa), the % wt content in PS and the % wt content in PHIC. Their molecular characteristics along with their micellar data by LALLS and DLS are given in Table 7, whereas the corresponding plots are provided in Figure 6 and Figure 7. The very low, even negative, A_2_ values for all samples by LALLS clearly indicate the reduced interactions between the polymers and the selective solvent. The degrees of association are unexpectedly high compared with the linear block copolymers having similar compositions and molecular weights. This is an indication that the specific macromolecular architecture promotes s strong intermicellar association, leading to the formation of clusters. It is evident from the LALLS plots that, upon increasing the concentration, there is a gradual increase in the molecular weight and that, only at relatively high concentrations, there is a plateau indicating that equilibrium is finally reached. The molecular weights, included in Table 7, reflect the results obtained from the higher concentration regime when equilibrium between micelles and clusters has been established. The block-graft terpolymer showed a very low degree of association due to both the low PS content and the specific complex macromolecular architecture, which prevents the organization of large multimolecular micelles.

The DLS measurements and the CONTIN analysis confirmed the hypothesis which was proposed by the LALLS data. Bimodal distributions were found in n-heptane in the case of the miktoarm star copolymers. Taking into account the DLS data in the common good solvent THF, it can be concluded that the major peak is attributed to small spherical and rather monodisperse micelles whereas the minority peak is attributed to the formation of clusters, which are not spherical and rather loose. The formation of clusters is due to the higher solution concentrations that are employed in order to achieve stable micellar structures. Upon increasing the concentration, there is sufficient space for intermicellar interactions, leading, to a small extent, to the formation of clusters. This behaviour is not observed in the case of the block-graft copolymer due to mainly the steric constraints imposed by the macromolecular architecture.

### 3.5. AFM Analysis

**Figure 8:**
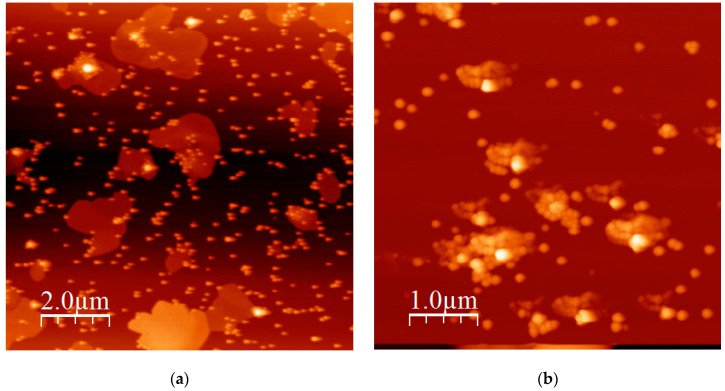
(**a**) Atomic force microscopy (AFM) height image of SH7.5/9/91 and (**b**) AFM height image of SH14/18/82.

To implement the conclusions drawn by static and dynamic light scattering measurements, AFM images were recorded. Characteristic images for the structures obtained by the SH7.5/9/91 and SH14/18/82 copolymers are given in Figure 8a,b, respectively. Mainly spherical compact micellar structures are observed in Figure 8a, and only a few isolated clusters can be traced in agreement with the conclusion drawn by DLS. The same picture is obvious in the case of the self-assemblies from the sample SH14/18/82. However, the spherical micellar structures are larger in this case and more extended clustering effects, through intermicellar interactions, are visible.

**Figure 9:**
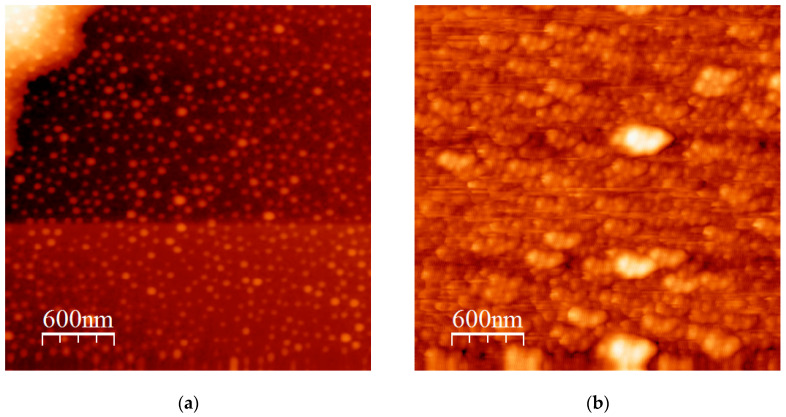
(**a**) AFM height image of HSH102/46/54 and (**b**) AFM height image of HSH187/41/59.

**Figure 10:**
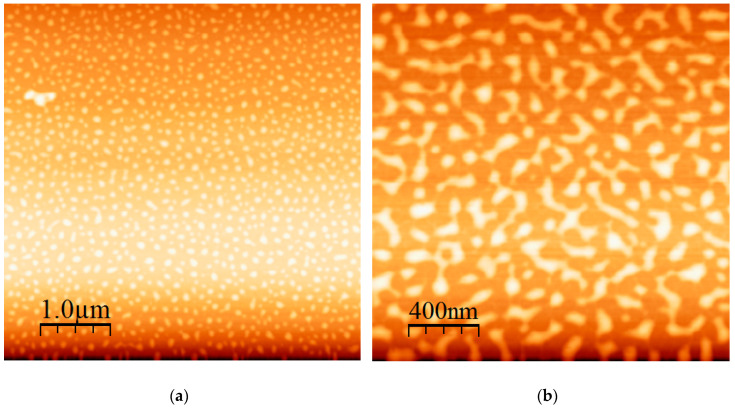
(**a**) AFM height image of HIH, c = 4 × 10^−4^ g/mL, and (**b**) AFM height image of HIH, c = 1 × 10^−3^ g/mL.

Spherical, relatively monodisperse, micelles are obtained in the case of the HSH102/46/54 sample, as shown in Figure 9a confirming the DLS data. Using a more concentrated solution of sample HSH187/41/59 in n-heptane, a similar picture was observed (Figure 9b). However, in this case, a small tendency for clustering is obvious. This is also facilitated by the rapid evaporation of the solvent in the spin coater in order to prepare the film for AFM observation. More interesting results were obtained in the case of the HIH triblock copolymer in n-dodecane. Small spherical structures are obtained from relatively dilute micellar solutions (Figure 10a). These are mainly unimolecular micelles constructed through the formation of loops from the end PHIC blocks. A limited number of intermicellar associates are observed under these experimental conditions. The situation is different using a higher initial micellar concentration, as shown in Figure 10b. The intermicellar associates dominate, leading to the appearance of non-spherical elongated structures.

**Figure 11:**
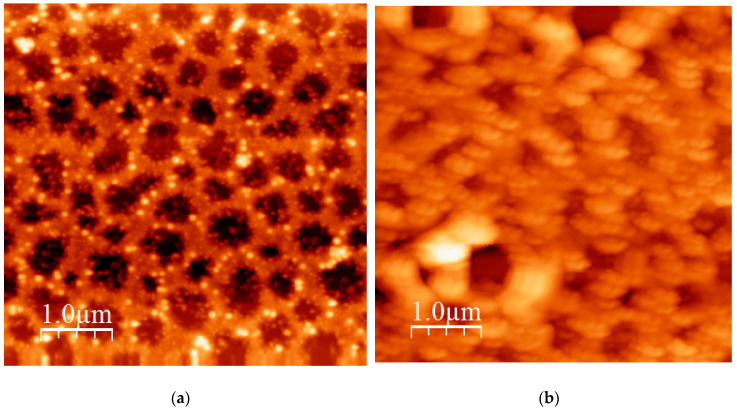
(**a**) AFM height image of HISIH and (**b**) AFM phase image of HISIH.

Another interesting result was obtained from the HSISH pentablock terpolymer micelles in n-heptane, as shown in Figure 11a,b. DLS measurements and the CONTIN analysis revealed the existence of equilibrium between spherical micelles and intermicellar associates. AFM images confirm that the initially formed spherical micelles are subjected to intermicellar fusion, leading to the formation of rather well-defined and extended networks. A similar behaviour was observed using core cross-linked micelles [78,79]. The initially formed spherical micelles were gradually transformed to cylindrical structures upon increasing to core component under the influence of external stimuli. Reversible morphological transitions from spherical to cylindrical micelles have been also observed upon changing the temperature [80]. An extremely similar behaviour was also observed when spherical micelles from block copolymers of PHIC with poly(2-vinyl pyridine), P2VP, formed in CHCl_3_ were subject to intermicellar fusion upon addition of a small amount of THF, which is a common good solvent for both blocks [66]. The result was the appearance of cylindrical micellar networks. In our case, similar phenomena are observed with the formation of an almost well-defined nanostructured micellar network. This result could be attributed to the macromolecular architecture, since the middle PI and the outer PHIC blocks are soluble in n-heptane and have to form the micellar corona, whereas the insoluble PS blocks, forming the micellar core, are the intermediate blocks. This architectural frustration leads to strong intermicellar interaction and finally to this characteristic network formation.

**Figure 12:**
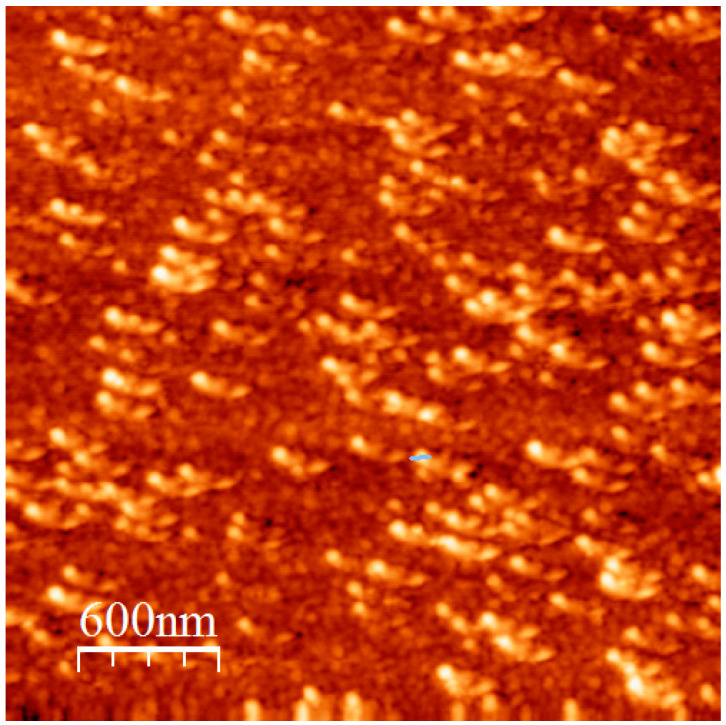
AFM height image of the PS(PHIC)_2_36/20/80.

**Figure 13:**
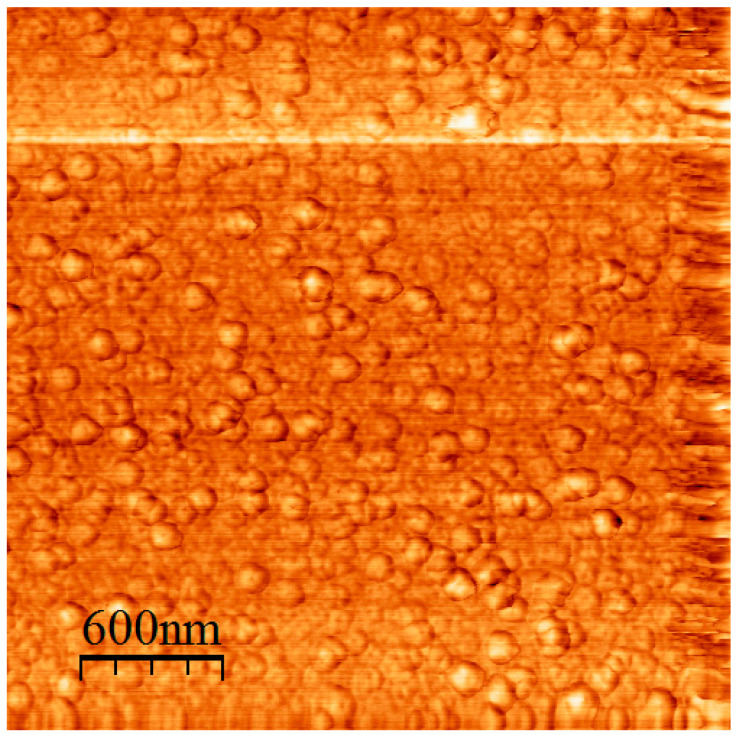
AFM height image of the block-graft copolymer.

**Figure 14:**
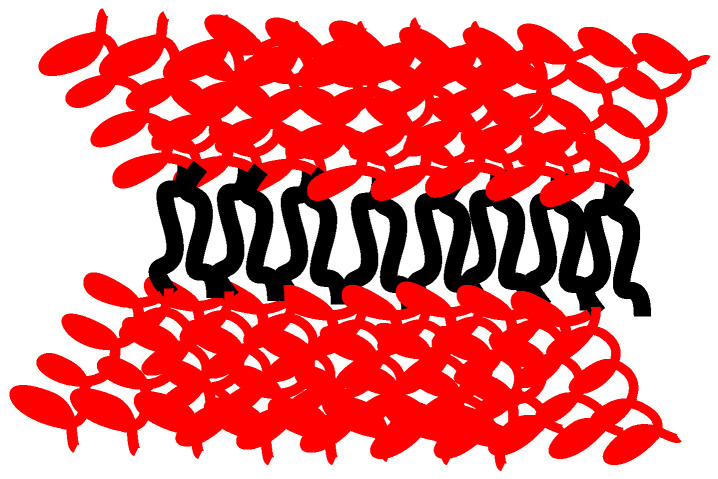
Schematic illustration of the cylindrical aggregates from the interaction of the initially formed spherical micelles.

A characteristic AFM image from the miktoarm star PS(PHIC)_2_36/20/80 is given in Figure 12, whereas a characteristic AFM image from the block-graft copolymer is given in Figure 13. The coexistence of small spherical micelles along with clusters is confirmed by AFM. The clusters are elongated and thus can be considered as small cylinders. This behaviour can be explained by an antiparallel arrangement of the star structures, which brings in close vicinity the insoluble PS blocks surrounded by the soluble PHIC chains (Figure 14). In the case of the block-graft copolymer, the DLS data were verified. Relatively uniform spherical micelles without clustering effects are obtained.

## 4. Conclusions

The micellization behaviour of various macromolecular architectures, involving diblock and triblock copolymers, pentablock terpolymers, miktoarm star copolymers and block-graft terpolymers, based on poly(hexyl isocyanate), PHIC, was studied in this work. n-Heptane, which is selective for the PHIC or polyisoprene (PI) chains, and n-dodecane, which is selective for the PI chains, were employed. The diblock copolymers form spherical micelles in equilibrium with clusters. The low PS content micelles behave as star-like structures, whereas the high PS content are actually crew-cut micelles. The HSH triblock copolymers form spherical, uniform and thermally stable micelles in n-heptane. The HIH micelles adopt a closed loop conformation in dilution solutions. However, upon increasing the concentration, intermicellar aggregates are formed, leading to elongated cylindrical-type structures. Clustering phenomena were obvious in the case of the pentablock terpolymers in both selective solvents. Of special interest is the HSISH terpolymer in n-heptane which formed an extended network from the intermicellar fusion of spherical micelles. The PS(PHIC)_2_ miktoarm star copolymers form spherical micelles in equilibrium with clusters, which appear to be linear arrangements of the spherical micelles. Finally, the block-graft terpolymer forms mainly unimolecular micelles. It is obvious that the manipulation of the macromolecular architecture leads to a complex micellar behaviour in selective solvents.

## Figures and Tables

**Table 1:** Molecular characteristics of the SH block copolymers and their micellar characteristics in n-heptane.

Sample	M_w_ × 10^−3 a^	Ð ^b^	%S, wt ^c^	A_2_^a^ × 10^4^	(M_w_ × 10^−3^)_mic_^a^	N_w_	D cm^2^/s ^d^ × 10^8^	k_D_^d^	R_1_^d^, nm	R_2_^d^, nm
SH14/3/97	14.0	1.32	3	−0.46	19.80	1.4	−78.99	66.7	4.40	-
SH7.5/9/91	7.5	1.29	9	0.49	24.30	3.2	58.02	42.6	9.41 (91.8%) ^e^	51.2 (8.2%) ^e^
SH19.5/11/89	19.5	1.22	11	2.00	161.0	8.3	9.75	667	55.9 (93.6%) ^e^	126.4 (6.4%) ^e^
SH14/18/82	14.0	1.18	18	−0.15	333.0	23.8	8.0	−12.5	68.2 (68.0%) ^e^	142.6 (32%) ^e^
SH121/90/10	121.0	1.19	90.4	−35.2	163,300	1350	2.0	−69.75	53.4	-
SH187/92/8	187.0	1.29	91.6	−0.79	247,800	1325	6.93	−470	79.4	-

^a^ By low-angle laser light scattering (LALLS), ^b^ by size exclusion chromatography (SEC) in tetrahydrofuran (THF), ^c^ by ^1^H NMR in CDCl_3_ at 25 °C, ^d^ by dynamic light scattering (DLS) in n-heptane, ^e^ by DLS in n-heptane.

**Table 2:** Structural characteristics of the SH block copolymers micelles in n-heptane.

Sample	N_B_	N_A_	R_h_, nm	R_h_/N_B_^2/3^	R_h_/N_B_^4/25^N_A_^3/5^	Dry Core		Swollen Core	
						R_c_, nm	A_c_, nm^2^	R_c_, nm	A_c_, nm^2^
SH14/3/97	4.04	106.9	4.40	1.73	0.21	0.61	3.31	0.76	5.26
SH7.5/9/91	6.49	53.74	9.41	2.70	0.64	0.94	3.46	1.18	5.48
SH19.5/11/89	20.62	136.65	55.9	7.43	1.80	1.88	5.37	2.37	8.53
SH14/18/82	24.23	90.39	68.2	8.14	2.74	2.83	4.22	3.56	6.70
SH121/90/10	1647.0	91.5	53.4	0.52	1.17	38.2	1.12	48.12	2.37
SH187/92/8	1051.8	123.7	79.4	0.57	1.35	44.1	0.97	55.68	1.17

**Table 3:** Molecular characteristics of the HSH and HIH triblock copolymers and their micellar characteristics in n-heptane and n-dodecane, respectively.

Sample	M_w_ × 10^−3 a^	Ð ^b^	%S (or I), wt ^c^	A_2_^a^× 10^5^	(M_w_ × 10^−3^)_mic_^a^	N_w_	THF			n-Heptane or n-Dodecane		
							D cm^2^/s ^d^ × 10^8^	k_D_^d^	R ^d^, nm	D cm^2^/s ^d^ × 10^8^	k_D_^d^	R ^d^, nm
HSH102/46/54	102.0	1.11	46.0	3.27	10,404	102	30.86	56.32	26.1	2.34	−56.40	70.9
HSH187/41/59	187.3	1.36	41.0	0.84	8147.6	43.5	48.48	37.50	16.6	2.88	−69.75	56.7
HIH	83.25	1.11	84.0	3.51	113.0	1.36	39.52	45.90	12.0	18.84 8.42	9.8 ^e^ −178.3	8.4(90%) ^e^ 18.8(10%)

^a^ By LALLS, ^b^ by SEC in THF, ^c^ by ^1^H NMR in CDCl_3_ at 25 °C, ^d^ by DLS in n-heptane and ^e^ by DLS in n-dodecane and CONTIN analysis.

**Table 4:** Viscometry results of the HSH triblock copolymers in n-heptane.

Sample	[η], dl/g	R_v_, nm	k_H_	R_v_/R_h_
HSH102/46/54	193.85	68.3	1.23	0.96
HSH187/41/59	92.17	49.3	0.63	0.87

**Table 5:** Structural characteristics of the HSH triblock copolymer micelles in n-heptane.

Sample	Dry Core		Swollen Core	
	R_c_, nm	A_c_, nm^2^	R_c_, nm	A_c_, nm^2^
HSH102/46/54	12.17	18.27	15.34	29.01
HSH187/41/59	10.80	33.72	13.61	53.52

**Table 6:** Molecular characteristics of the HSISH and HISIH pentablock terpolymers and their micellar characteristics in n-heptane and n-dodecane.

Sample	Ð ^a^	%S, wt ^b^	%I wt ^b^	%H wt ^b^	Solvent	M_w_ × 10^−3 c^	A_2_^c^	R_h_^d^, nm	k_D_^d^	N_w_
HSISH	1.25	46	41	13	THF	99.250	5.2 × 10^−4^	13.6	53.6	-
					C7	21,600.00	1.68 × 10^−6^	32.5 62.8(54%) ^e^	30.1–32.2	217.6
					C12	62,000.00	1.55 × 10^−7^	39.0 93.2(53%) ^e^	38.4–39.6	624.7
HISIH	1.32	43	32	25	THF	169.00	0.15	17.0	42.7	-
					C7	717.00	0.12	47.1 109.4(49%) ^e^	38.3 0.05	4.24
					C12	1492.30	0.26	32.0 78.4(58%) ^e^	12.7 4.7	8.83

^a^ By SEC in THF, ^b^ by ^1^H NMR in CDCl_3_ at 25 °C, ^c^ by LALLS, ^d^ by DLS in n-heptane or n-dodecane and ^e^ by CONTIN analysis.

**Table 7:** Molecular characteristics of the PS(PHIC)_2_ miktoarm star copolymers and the PS-b-(PI-g-PHIC)block graft terpolymer and their micellar characteristics in n-heptane.

Sample	M_w_ × 10^−3 a^	Ð ^b^	%S/H/I, wt ^c^	A_2_^a^ × 10^−3 a^	(M_w_ × 10^−3^)_mic_^a^	N_w_	THF		n-heptane		
							D ^d^ cm^2^/s ^d^ × 10^7^	R_h_^d^, nm	D ^e^ cm^2^/s ^d^ × 10^7^	R_h1_^e^, nm	R_h2_^e^, nm
PS(PHIC)_2_30/17/83	29.7	1.18	17.0/83.0/0	0.1	5000	168	5.74	8.3	2.53	21.6 (86.0%)	144.0 (14%)
PS(PHIC)_2_33/17/83	32.9	1.13	17.1/82.9/0	−1.5	1000	30.4	2.65	7.8	2.68	20.4 (90.5%)	400.9 (9.5%)
PS(PHIC)_2_36/20/80	36.4	1.13	20.4/79.6/0	−1.1	333.0	9.1	3.39	14.1	1.75	31.1 (75.0%)	62.7 (25.0%)
PS-b-(PI-g-PHIC)	91.6	1.33	14.0/82.0/4.0	−0.80	103.0	1.1	3.99	11.9	2.00	27.2	-

^a^ By LALLS, ^b^ by SEC in THF, ^c^ by ^1^H NMR in CDCl_3_ at 25 °C, ^d^ by DLS in THF and ^e^ by DLS in n-heptane and CONTIN analysis.

## Supplementary Materials

The following are available online at https://www.mdpi.com/2073-4360/12/8/1678/s1, Figure S1: LALLS plot for the sample SH187/92/8; Figure S2: LALLS plot for the sample SH121/90/10; Figure S3: DLS plot for sample SH187/92/8; Figure S4: DLS plot for sample SH14/18/82; Figure S5: DLS plot for sample HIH; Figure S6: Huggins (_▪_) and Kraemer (●) plots for sample SHS102/46/54; Figure S7: Huggins (_▪_) and Kraemer (●) plots for sample SHS187/41/59; Figure S8: AFM height image for sample PS(PHIC)_2_36/20/80.
