# Supplementary material for: Micellization Behaviour of Linear and Nonlinear Block Copolymers Based on Poly(n-hexyl isocyanate) in Selective Solvents
Source: Polymers (Basel). 2020 Jul 28;12(8):1678. doi: 10.3390/polym12081678 (PMC7465233; doi:10.3390/polym12081678)
Supplement: Supplementary file 1 [file polymers-12-01678-s001.pdf]

Micellization behavior of linear and non-linear block copolymers based on poly(n-hexyl isocyanate) in selective solvents.

Aggelos Vazaios, Athanasios Touris, Mikel Echeverria, Georgia Zorba and Marinos Pitsikalis*

Industrial Chemistry Laboratory, Department of Chemistry, National and Kapodistrian University of Athens, Panepistimiopolis Zografou, 15771 Athens Greece

Supporting Information Section

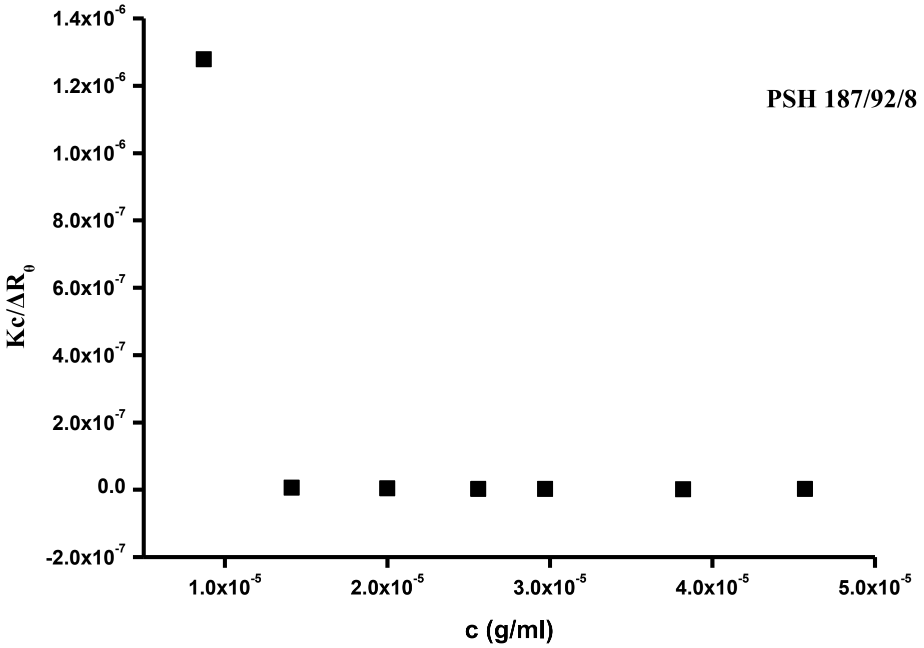

Figure S1: LALLS plot for the sample SH187/92/8

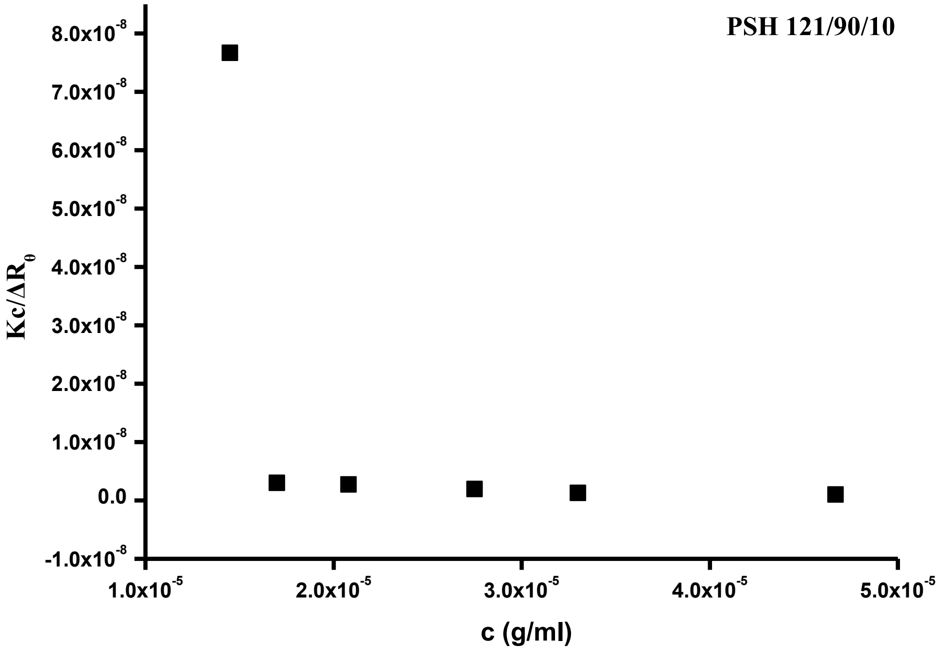

Figure S2: LALLS plot for the sample SH121/90/10

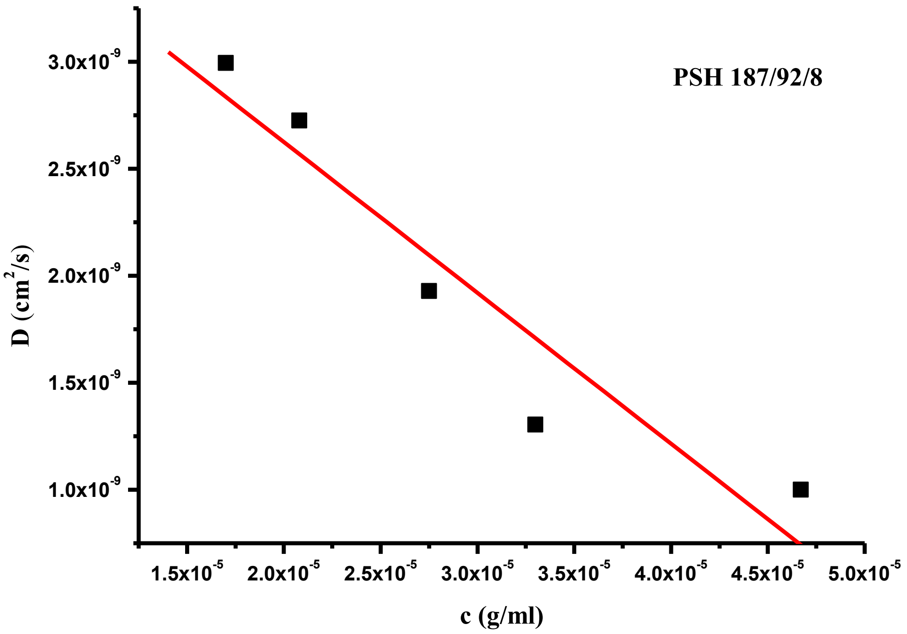

Figure S3: DLS plot for sample SH187/92/8

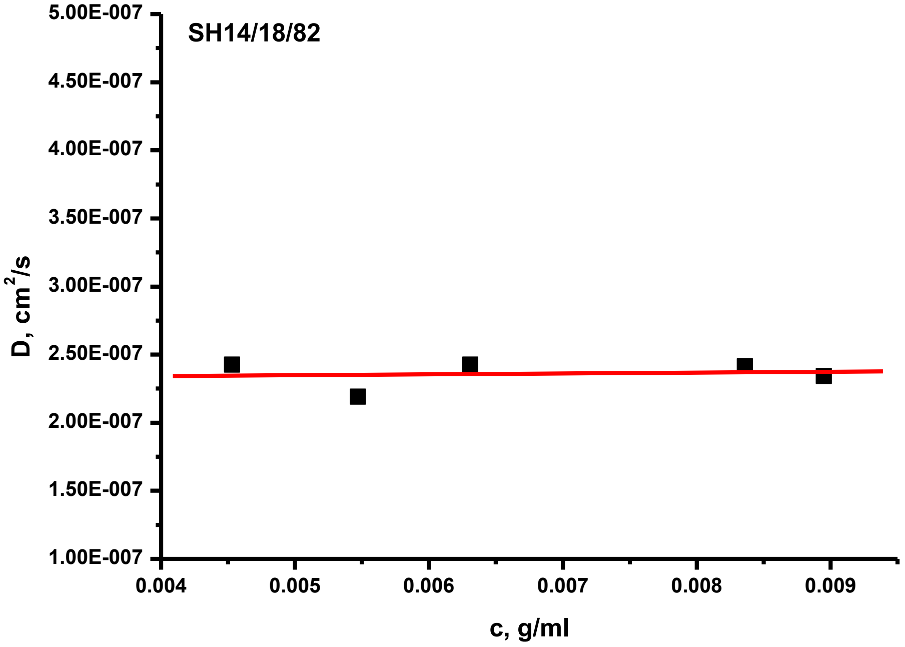

Figure S4: DLS plot for sample SH14/18/82

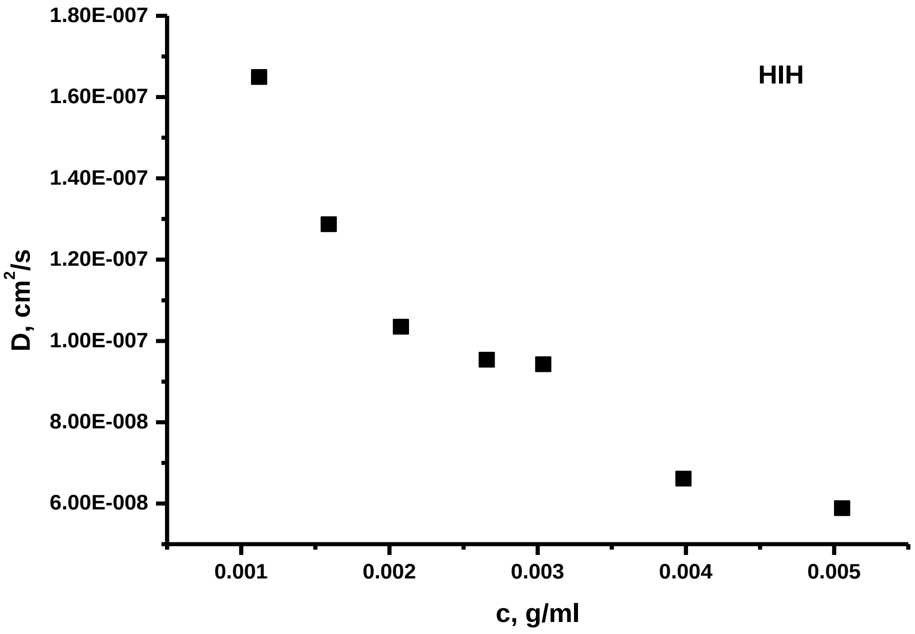

Figure S5: DLS plot for sample HIH

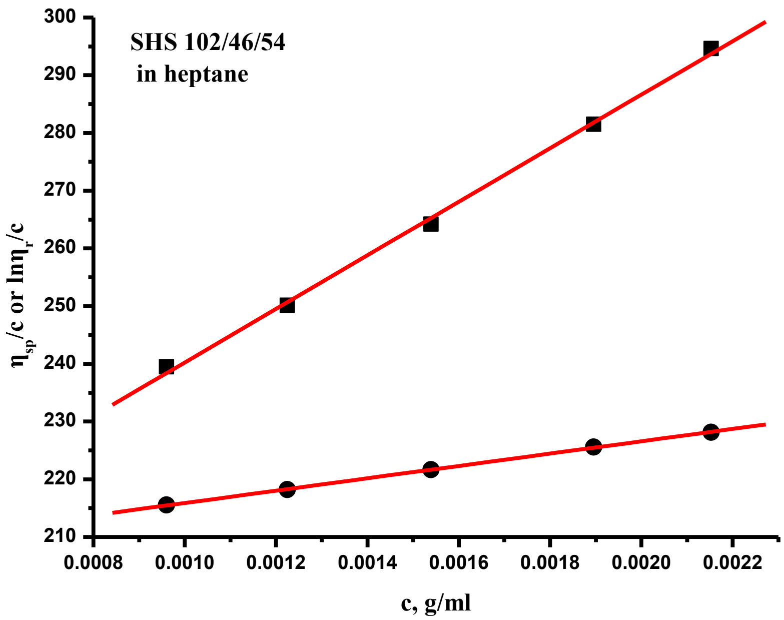

Figure S6: Huggins (▪) and Kraemer (●) plots for sample SHS102/46/54

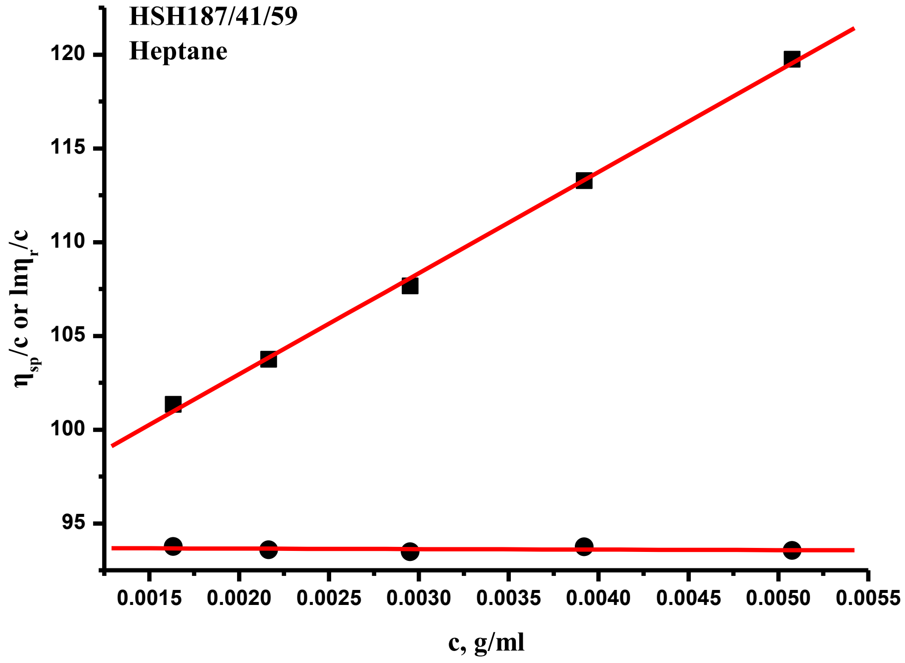

Figure S7: Huggins (▪) and Kraemer (●) plots for sample SHS187/41/59

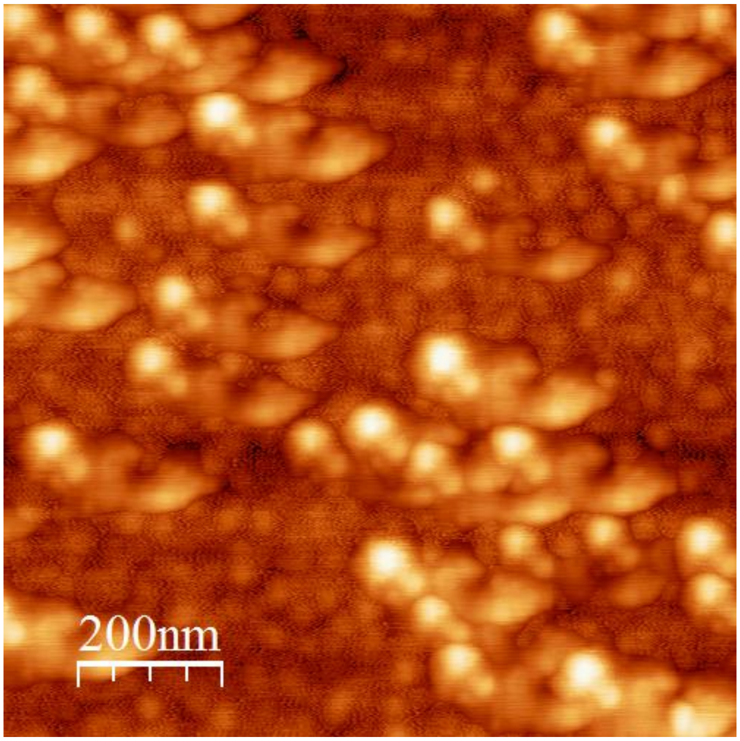

Figure S8: AFM height image for sample $PS(PHIC)_2$36/20/80
